# Structural analysis of *Helicobacter pylori* glutamate racemase in a monoclinic crystal form

**DOI:** 10.1107/S2053230X26008782

**Published:** 2026-10-09

**Authors:** Maria Spiliopoulou, Eike C. Schulz

**Affiliations:** a University Medical Center Hamburg-Eppendorf (UKE) Hamburg Germany; b Institute of Nanostructure and Solid State Physics, University of Hamburg, Germany; c Max Planck Institute for the Structure and Dynamics of Matter, Hamburg, Germany; University of São Paulo, Brazil

**Keywords:** glutamate racemases, dimers, monoclinic symmetry

## Abstract

Glutamate racemase (MurI) catalyzes the stereochemical interconversion of l-glutamate and d-glutamate, a key element of bacterial peptidoglycan biosynthesis. In this study, we present the crystal structure of *Helicobacter pylori* glutamate racemase at 1.43 Å resolution and with monoclinic symmetry, as in previously reported models, but with different unit-cell parameters. The present model contains a single dimer in the asymmetric unit and retains the previously described head-to-head dimer arrangement. Comparative analysis of crystal packing reveals that the conserved dimeric assembly frequently adopts similar packing motifs, while differences in the relative arrangement of these dimeric arrays result in altered crystal packing and variations in unit-cell parameters. The monomeric fold and active-site architecture remain conserved and are consistent with the catalytic features described for bacterial glutamate racemases. This structure provides an updated, high-resolution structural model for *H. pylori* glutamate racemase and highlights the variability of crystal-packing arrangements within related monoclinic crystal forms.

## Abstract 2

The crystal structure of *H. pylori* MurI is reported at 1.43 Å resolution.

## 1. Introduction

Glutamate racemase (MurI) is a cofactor-independent enzyme that catalyzes the reversible stereochemical conversion of l-glutamate and d-glutamate, an essential component of bacterial peptidoglycan (murein). The production of d-glutamate is required for cell-wall biosynthesis, and inhibition of MurI disrupts peptidoglycan assembly, making the enzyme an attractive target for antibacterial drug development (Grishin *et al.*, 2020 ▸; Keating, 2013 ▸). In addition to its metabolic role, MurI has been reported to exhibit secondary functions, such as inhibition of DNA gyrase, highlighting its functional versatility (Fisher, 2008 ▸).

MurI enzymes catalyze racemization through a cofactor-independent two-base mechanism involving deprotonation of the substrate at the α-carbon followed by reprotonation on the opposite face. This reaction is mediated by conserved cysteine residues that act as general acid–base catalysts, and MurI proteins typically consist of two domains (α, β) that form an active site at their interface, with catalytic residues positioned to facilitate stereoinversion of the bound substrate (Ruzheinikov *et al.*, 2005 ▸; Grishin *et al.*, 2020 ▸).

Glutamate racemase from *Helicobacter pylori* adopts the standard MurI fold and functions as a homodimer, which represents the biologically active form of the enzyme. The dimer interface contributes to enzyme stability and plays a role in shaping the active-site environment with a head-to-head shape (Fisher, 2008 ▸; Lundqvist *et al.*, 2007 ▸). Recent studies have further demonstrated that dimerization is functionally coupled to enzyme dynamics, with inter-subunit communication playing a critical role in catalysis. In particular, the identification of a cryptic allosteric pocket in *H. pylori* glutamate racemase has revealed that the binding of small-molecule inhibitors can disrupt coupled motions between monomers, thereby impairing catalytic activity (Chheda *et al.*, 2021 ▸). These findings highlight the importance of dynamic inter­actions across the dimer interface and provide a mechanistic basis for allosteric regulation of MurI. Previous structural studies of *H. pylori* MurI have provided insight into substrate binding, catalytic mechanism and inhibition, consistently reporting dimeric assemblies in the crystal structures (Fisher, 2008 ▸; Lundqvist *et al.*, 2007 ▸).

In this study, we present a crystallographic analysis of *H. pylori* glutamate racemase, contributing to the further characterization of its molecular organization and dimer interface. Moreover, time-resolved crystallography (TRX) benefits from highly homogeneous crystals with sufficient solvent content to facilitate ligand diffusion and synchronized reaction initiation. In this context, crystallization conditions for *H. pylori* glutamate racemase were explored to identify crystal forms with improved properties. Of particular interest is the arrangement of dimers with respect to each other within the crystal. This work provides additional structural information that contributes to understanding the variability in crystal packing and unit-cell organization in MurI enzymes.

## 2. Materials and methods

### 2.1. Protein purification and crystallization

The *H. pylori* MurI gene was cloned in pET-28a(+) vector (GenScript) and expressed in Arctic Express *Escherichia coli* cells grown in LB medium supplemented with 20 µg ml^−1^ gentamycin and 50 µg ml^−1^ kanamycin as the overnight starting culture at 37°C. For the large-scale culture, LB medium was supplemented with 50 µg ml^−1^ kanamycin and the cells were incubated at 30°C for 3 h (OD_600_ ≃ 0.7). Protein expression was induced by the addition of isopropyl β-d-1-thiogalactopyranoside to a final concentration of 0.001 *M* and the cells were further incubated at 13°C for 24 h. The cells were harvested by centrifugation (7500*g*, 20 min, 4°C) and the pellets were stored at −20°C until purification. The pellets were resuspended in lysis buffer [0.05 *M* Tris pH 8.0, 0.15 *M* NaCl, 5%(*v*/*v*) glycerol] and sonicated for lysis. The protein was purified by nickel-affinity chromatography using 0.05 *M* Tris–HCl, 0.15 *M* NaCl, 0.001 *M* DTT, 0.002 *M* ATP-Mg^2+^ pH 8.0 supplemented with 0.005 and 0.5 *M* imidazole pH 8.0 as the binding and elution buffer, respectively. Size-exclusion chromatography followed with a final buffer consisting of 0.2 *M* ammonium acetate pH 7.4, 0.005 *M*dl-glutamate, 0.001 *M* DTT (Lundqvist *et al.*, 2007 ▸). Crystallization was performed in sitting drops by mixing 1 µl 10 mg ml^−1^ protein, 0.8 µl crystallization buffer and 0.2 µl seeds and equilibrating against 100 µl reservoir consisting of 0.1 *M* Tris pH 8.5, 0.1 *M* MgSO_4_, 20%(*w*/*v*) PEG 4000.

### 2.2. X-ray data collection

Single-crystal rotation datasets were collected on beamline P13 at PETRA-III, DESY, Hamburg (Cianci *et al.*, 2017 ▸) at the EMBL Hamburg unit using an EIGER 16M detector. Data-collection and structure-refinement parameters are summarized in Table 1 ▸.

### 2.3. Data processing

Diffraction data were automatically processed with *AUTOPROC* using *STARANISO* (Vonrhein *et al.*, 2011 ▸; Tickle *et al.*, 2018 ▸). The diffraction limits were determined automatically using the anisotropic resolution cutoff, which estimates the local diffraction limit from the statistical significance of the data in different directions of reciprocal space, based on the local mean *I*/σ(*I*), with corresponding CC_1/2_ statistics. This anisotropic truncation retains statistically significant reflections, and the reported completeness values correspond to the anisotropically truncated dataset and are not directly comparable with conventional spherical completeness values. Molecular replacement was conducted in *Phaser* (McCoy *et al.*, 2007 ▸) using PDB entry 2jfy (Lundqvist *et al.*, 2007 ▸) as the starting model. Structures were refined using iterative cycles of *REFMAC* (Vagin *et al.*, 2004 ▸) and *Coot* (Emsley *et al.*, 2010 ▸). *B* factors were refined isotropically. Molecular images were generated in *PyMOL* (Schrödinger, 2015 ▸) and *UCSF Chimera* (Pettersen *et al.*, 2004 ▸). Molecular interactions were inferred from parsed PDB structures using distance heuristics and projected into a 2D plane, with residues spatially organized around the central ligand. The resulting visualizations were rendered as scalable vector graphics (SVG), depicting atoms, bonds and labels, with dashed lines used to represent non­covalent interactions.

### 2.4. Calculation of C^α^ r.m.s.d.

In order to quantify and visualize the changing global structural differences, a pairwise backbone root-mean-square deviation (C^α^ r.m.s.d.; Kabsch, 1976 ▸; Carugo & Pongor, 2001 ▸) was calculated and represented as a categorical heatmap. Before calculating the r.m.s.d. for a pair, the two structures were aligned via singular value decomposition (SVD; Brunton & Kutz, 2019 ▸) using the Biopython package *Bio.SVDSuperimposer* in order to minimize the resulting r.m.s.d. value (Schulz *et al.*, 2025 ▸).

### 2.5. Solvent-channel analysis

Solvent-channel analysis was performed using *MAP_CHANNELS* (Juers & Ruffin, 2014 ▸). The program computes a distance map from the atomic coordinates and performs a multiscale cluster analysis to identify connected solvent channels and determine their dimensionality (3D, 2D or 1D). The maximum channel radius was defined as the radius of the largest spherical probe capable of continuously traversing the crystal lattice in the corresponding dimensionality.

### 2.6. Crystal-contact identification

Crystal contacts were analysed using the *CCP*4 *CONTACT* software (Agirre *et al.*, 2023 ▸). Symmetry-related molecules were generated according to the crystallographic symmetry operators, and intermolecular contacts between atoms from neighbouring asymmetric units were identified based on interatomic distance criteria. Contacts within a defined distance cutoff of 3.2 Å were used to compare the symmetry-related packing interactions between the different *H. pylori* MurI crystal forms.

### 2.7. Crystal-packing analysis and visualization

Crystal packing was analysed and rendered with a custom Python script built on *PyMOL* 3.1 (Schrödinger, 2015 ▸) and the *gemmi* library version 0.7.5 (Wojdyr, 2022 ▸). Atomic coordinates, unit-cell parameters and space-group symmetry were read directly from the deposited coordinate files. For each structure the reference asymmetric unit was taken as the deposited coordinates. Symmetry-equivalent molecules were generated by applying the crystallographic symmetry operations of the space group, combined with unit-cell translations, as Cartesian transformations constructed from the *gemmi* orthogonalization matrices.

#### 2.7.1. Figure generation

For each structure, three complementary representations were produced, and two of them are used for the current study: (i) one-dimensional arrays of molecules along each crystallographic axis (*a*, *b*, *c*), (ii) two-dimensional packing monolayers in the *ab*, *ac* and *bc* planes, restricted to the single layer of molecules containing the reference, and (iii) a three-dimensional view of the reference asymmetric unit together with its contacting symmetry mates within the outlined unit cell. The reference molecule is drawn in blue and symmetry-related molecules in grey; residues forming crystal contacts are shown as spheres, coloured orange for the reference and green for the symmetry mate, and polar contacts are drawn as dashed lines. All colours are taken from the colour-blind-safe palette of Wong (2011 ▸). Crystallographic axes are drawn as labelled arrows.

#### 2.7.2. Transparency statement on the use of artificial intelligence

The analysis and visualization script described above was developed with the Claude code command-line interface (model identifier claude-opus-4-8). The model was used for code generation, debugging and drafting under the direct supervision of the authors. All generated code was executed, inspected and verified by the authors, and all parameter choices, scientific content and interpretations were checked by and remain the responsibility of the authors. No sensitive or personal data were provided to the model.

## 3. Results and discussion

### 3.1. Crystal-quality optimization

The initial crystallization condition was based on a previously reported condition for *H. pylori* glutamate racemase consisting of 0.1 *M* Tris pH 8.5, 0.2 *M* MgSO_4_, 25%(*w*/*v*) PEG 4000 (Lundqvist *et al.*, 2007 ▸). This condition was subsequently optimized by varying the pH (from 7.0 to 8.5) and the MgSO_4_ (from 0.1 to 0.4 *M*) and PEG 4000 (from 10 to 25%) concentrations to improve the crystal quality and identify conditions that yielded well diffracting crystals.

The morphology of the crystals was strongly affected by pH and PEG concentration variations (Table 2 ▸), regardless of the MgSO_4_ concentration. Low-pH (<8.0) and low-PEG (<20%) conditions yielded long needles, while the combination of higher pH values (≥8.0) and higher PEG (≥20%) concentration resulted in shorter, thicker needles accompanied by altered unit-cell parameters.

#### 3.1.1. Seeding

**Figure 1:**
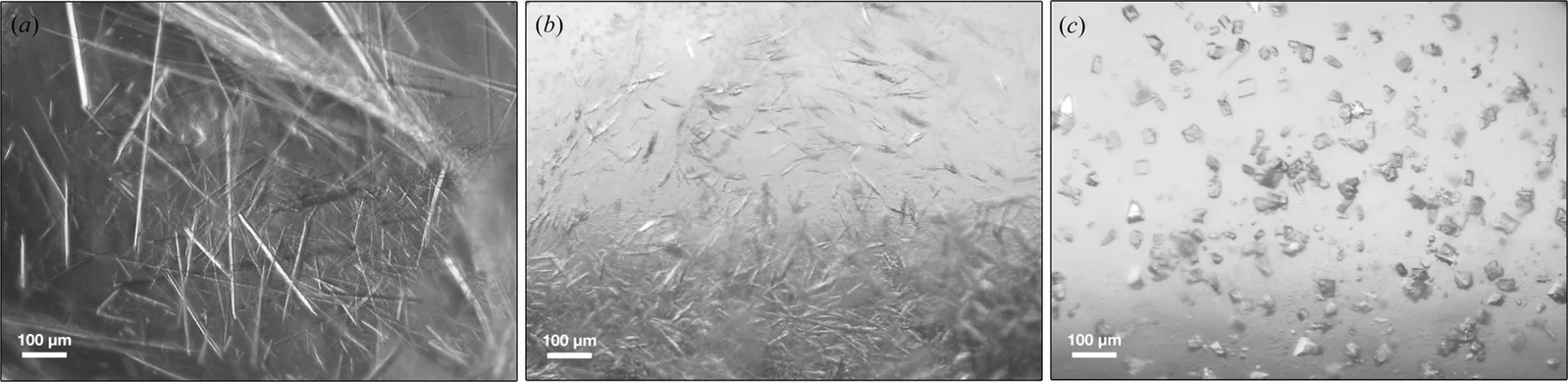
Photographs of MurI protein crystals under different crystallization conditions. (*a*) 0.4 *M* MgSO_4_, 10% PEG, 0.1 *M* Tris pH 8.5; (*b*) 0.1 *M* MgSO_4_, 20% PEG, 0.1 *M* Tris pH 8.5; (*c*) 0.1 *M* MgSO_4_, 20% PEG, 0.1 *M* Tris pH 8.5, seeding.

To further explore the influence of microseeding on crystal growth in sitting drops, a seed stock was prepared from the short thick needles, which were more stable and did not fragment during manipulation. This seed stock was then introduced into selected conditions from the full screen of varying salt/PEG concentration and pH (mixing 1 µl 10 mg ml^−1^ protein solution, 0.8 µl crystallization buffer and 0.2 µl seeds). Under high-pH and high-PEG conditions, seeding produced crystals with a markedly different morphology (Fig. 1 ▸): the crystals were more homogeneous in both shape and size, while maintaining the same unit-cell parameters as the original short, thick needles. These observations indicate that seeding can stabilize crystal growth under specific conditions, leading to improved uniformity without altering the lattice parameters. This could be of particular interest, especially for expanding this study and using these crystals in TRX experiments (Schulz *et al.*, 2022 ▸).

### 3.2. The monoclinic structural model of MurI

**Figure 2:**
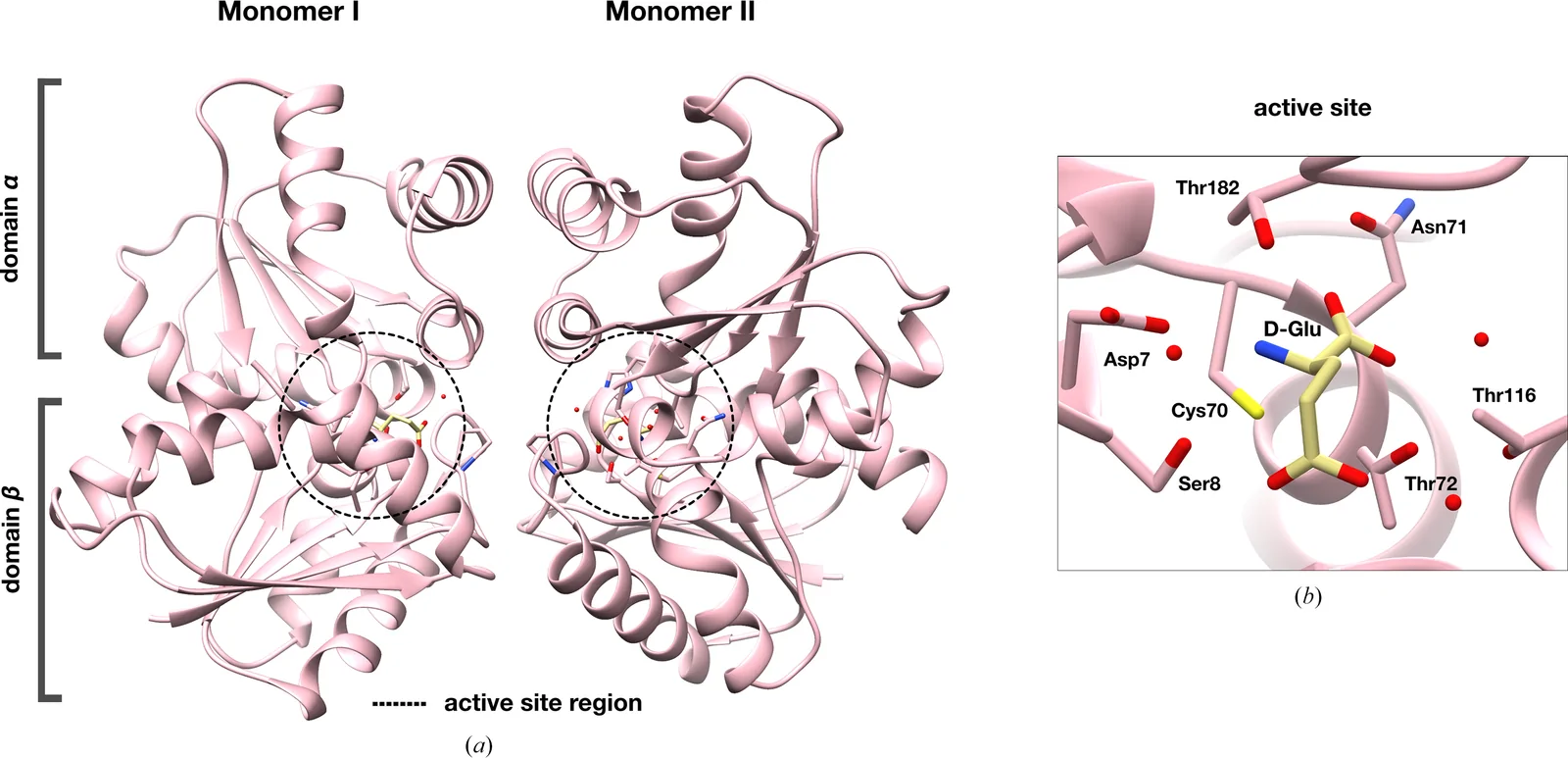
Cartoon representation of the MurI crystal structure. (*a*) The dimer arrangement with the active-site regions indicated in circles. (*b*) Close-up of the active site and the surrounding amino acids.

The crystal structure of *H. pylori* glutamate racemase was determined in a monoclinic space group, with unit-cell parameters *a* = 59.09, *b* = 72.95, *c* = 70.16 Å, β = 113.59° (Table 1 ▸). The asymmetric unit contains one homodimer, consistent with previously reported structures (Lundqvist *et al.*, 2007 ▸). The overall fold of each monomer corresponds to the characteristic architecture of glutamate racemases, consisting of two domains (α and β; Fig. 2 ▸).

Previous analyses have shown that MurI glutamate racemases show seemingly species-specific oligomerization and subunit organization. While *E. coli* MurI forms a monomer, other bacterial MurI glutamate racemases form dimers. In enterococci (*Enterococcus faecalis* and *E. faecium*) and *Staphylococcus aureus* these arrange into tail-to-tail organized dimers. On the contrary, *H. pylori* MurI adopts a head-to-head dimer organization, which is also conserved in our structure (Lundqvist *et al.*, 2007 ▸).

**Figure 3:**
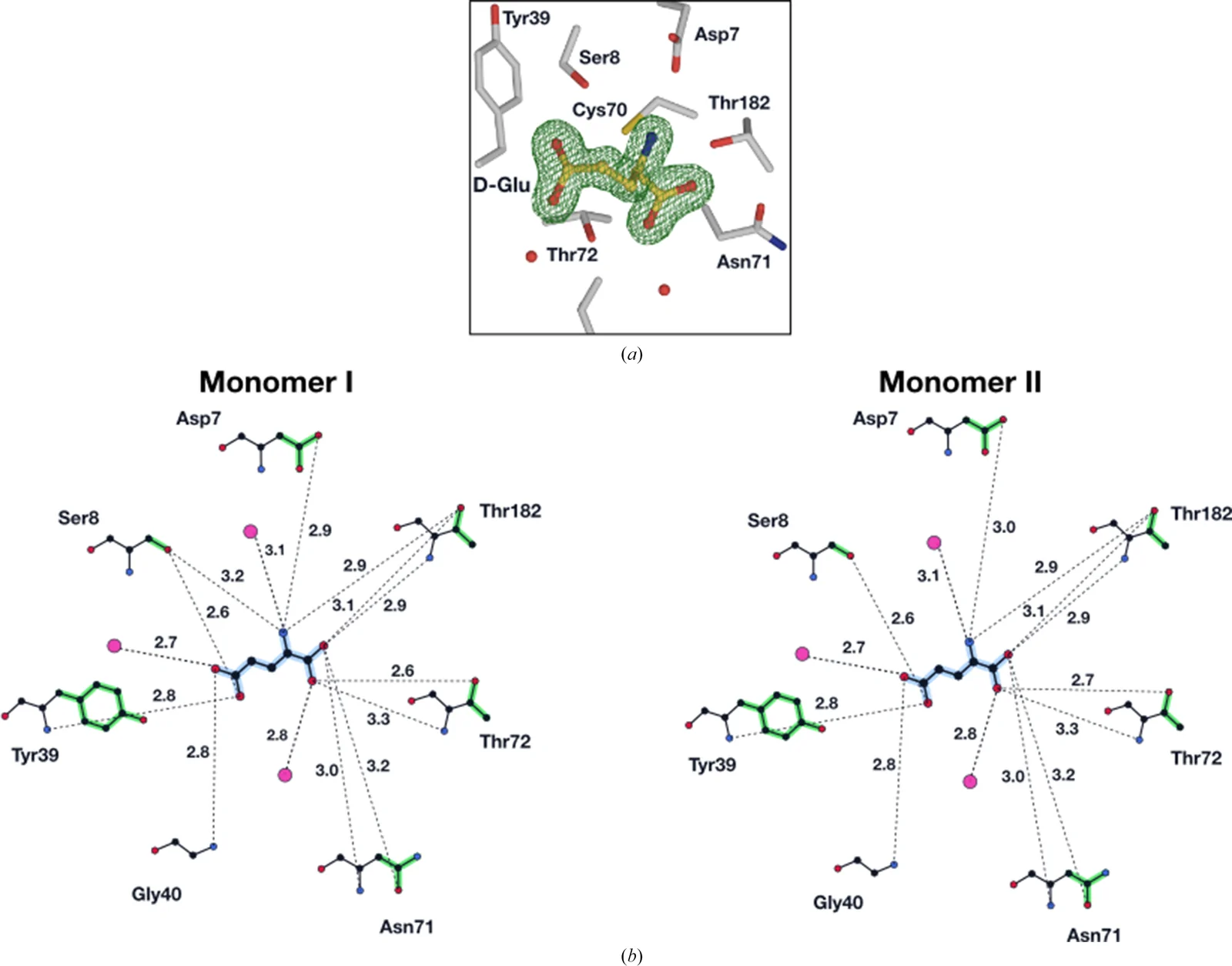
Molecular interactions of d-glutamate. (*a*) 3D representation of d-glutamate and the amino acids in the active site. An omit *mF*_o_ − *DF*_c_ map (Terwilliger *et al.*, 2008 ▸) contoured at 1.5 r.m.s.d. is displayed as a green mesh. (*b*) 2D representation of the hydrogen bonds that each d-glutamate molecule in the MurI dimer forms to the surrounding amino acids and water molecules. d-Glutamate (centre) is highlighted in blue; hydrogen bonds are shown as dashed lines with lengths marked; amino acids are marked in three-letter code with side chains highlighted in green; water molecules are shown as pink circles.

**Figure 4:**
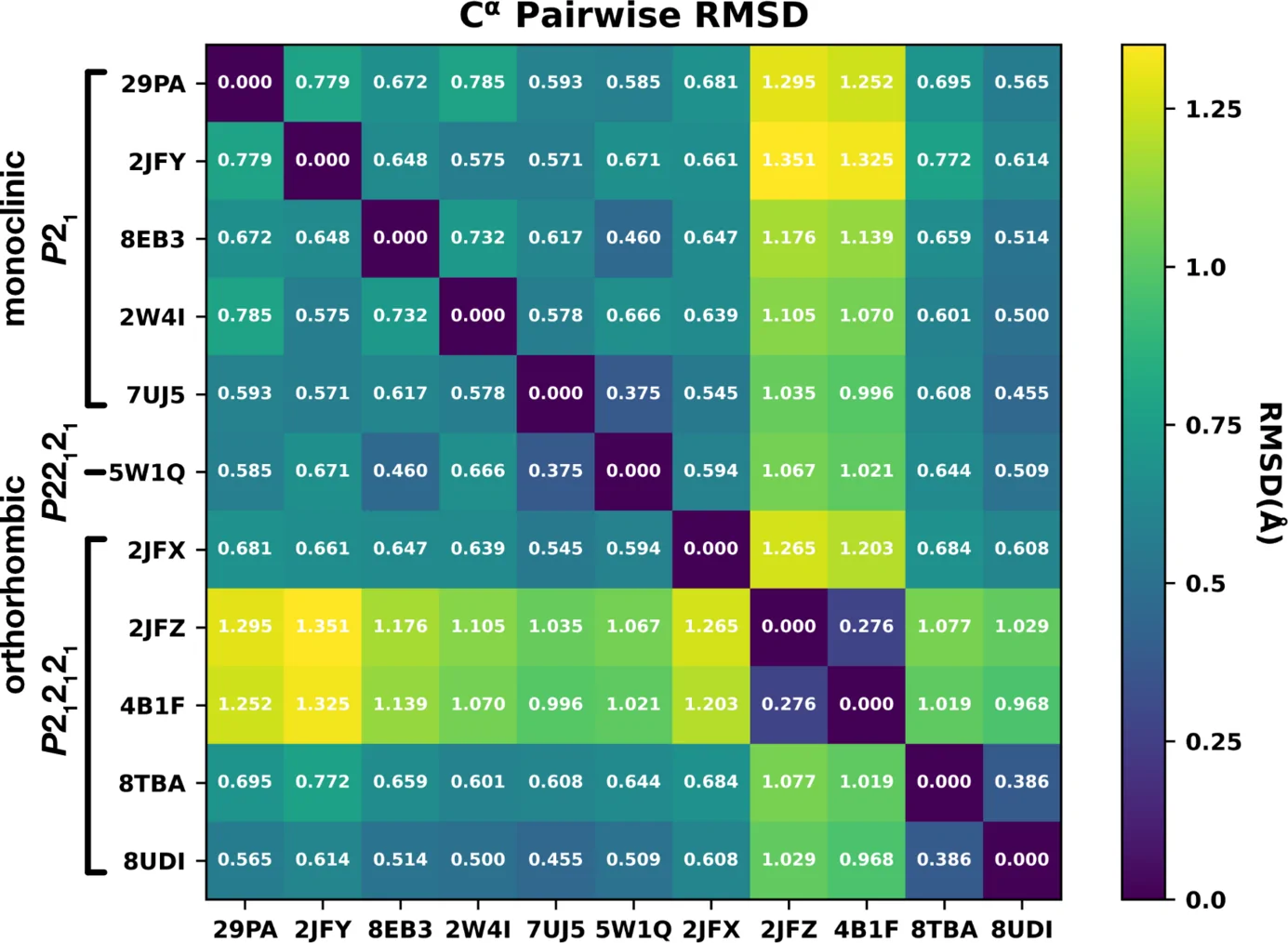
C^α^ r.m.s.d. analysis visualizing structural deviations among *H. pylori* MurI structures.

The data extend to a resolution of 1.43 Å, resulting in a clear electron-density map, which allowed precise modelling of the overall protein and d-glutamate in both active sites. The ligand is positioned within the catalytic pocket and interacts with the surrounding amino acids (Fig. 3 ▸). No major conformational differences are observed compared with earlier models; thus, the refined structure represents a typical example of the enzyme in its dimeric, product-bound form (Fig. 4 ▸).

Beyond accurate modelling of the protein and bound d-glutamate, the improved resolution allows a more confident placement of ordered solvent molecules in the active site. Comparison of the active-site interactions across all deposited *H. pylori* MurI structures (Supplementary Figs. S1, S2 and S3) shows that the catalytic architecture and the principal protein–ligand interactions are highly conserved.

### 3.3. Structural comparison and crystal-packing analysis

Structural comparison with previously reported models of *H. pylori* glutamate racemase reveals a conserved dimer organization among structures crystallizing in space group *P*2_1_. The deposited structures adopt the characteristic head-to-head dimer arrangement, in which the active sites are oriented towards the internal face of the dimer interface (Lundqvist *et al.*, 2007 ▸). Despite differences in crystallization conditions and asymmetric unit composition, the overall quaternary assembly is preserved, with all structures containing the same chain arrangement within the dimer.

To compare our structure (PDB entry 29pa) with previously reported *H. pylori* MurI structures we used the global C^α^ r.m.s.d. analysis (Kabsch, 1976 ▸; Carugo & Pongor, 2001 ▸) by superimposing 490–510 C^α^ atoms per model pair (Fig. 4 ▸). Our structure exhibits the same dimeric organization as the deposited models, with differences primarily arising from variations in crystal packing rather than changes in the quaternary assembly.

Across the analysed structures, the C^α^ r.m.s.d. values remained below 1.3 Å, including comparisons between structures belonging to different space groups, and below 0.8 Å for structures in space group *P*2_1_, demonstrating a high degree of structural conservation despite substantial differences in crystal-packing arrangements. Two structures (PDB entries 2jfz and 4b1f) showed comparatively higher r.m.s.d. values; however, these models displayed high structural similarity to each other. Both structures correspond to *H. pylori* MurI bound to an inhibitor and d-glutamate, suggesting that ligand binding may contribute to the observed conformational differences. Thus, while crystal-packing variations have limited effects on the global architecture of MurI, interactions with bound ligands may introduce additional structural rearrangements.

To evaluate the relationship between crystal packing and lattice properties, the refined model was compared with previously reported *H. pylori* MurI structures (Table 3 ▸), with particular emphasis on the three of them that share the same space group (*P*2_1_) and the same number of dimers in the asymmetric unit (PDB entries 29pa, 2jfy and 8eb3). Although these structures differ in unit-cell dimensions, they exhibit comparable solvent content and lattice volumes, indicating that optimization of the crystallization conditions preserved the overall packing density while improving the crystal morphology and diffraction quality.

**Figure 5:**
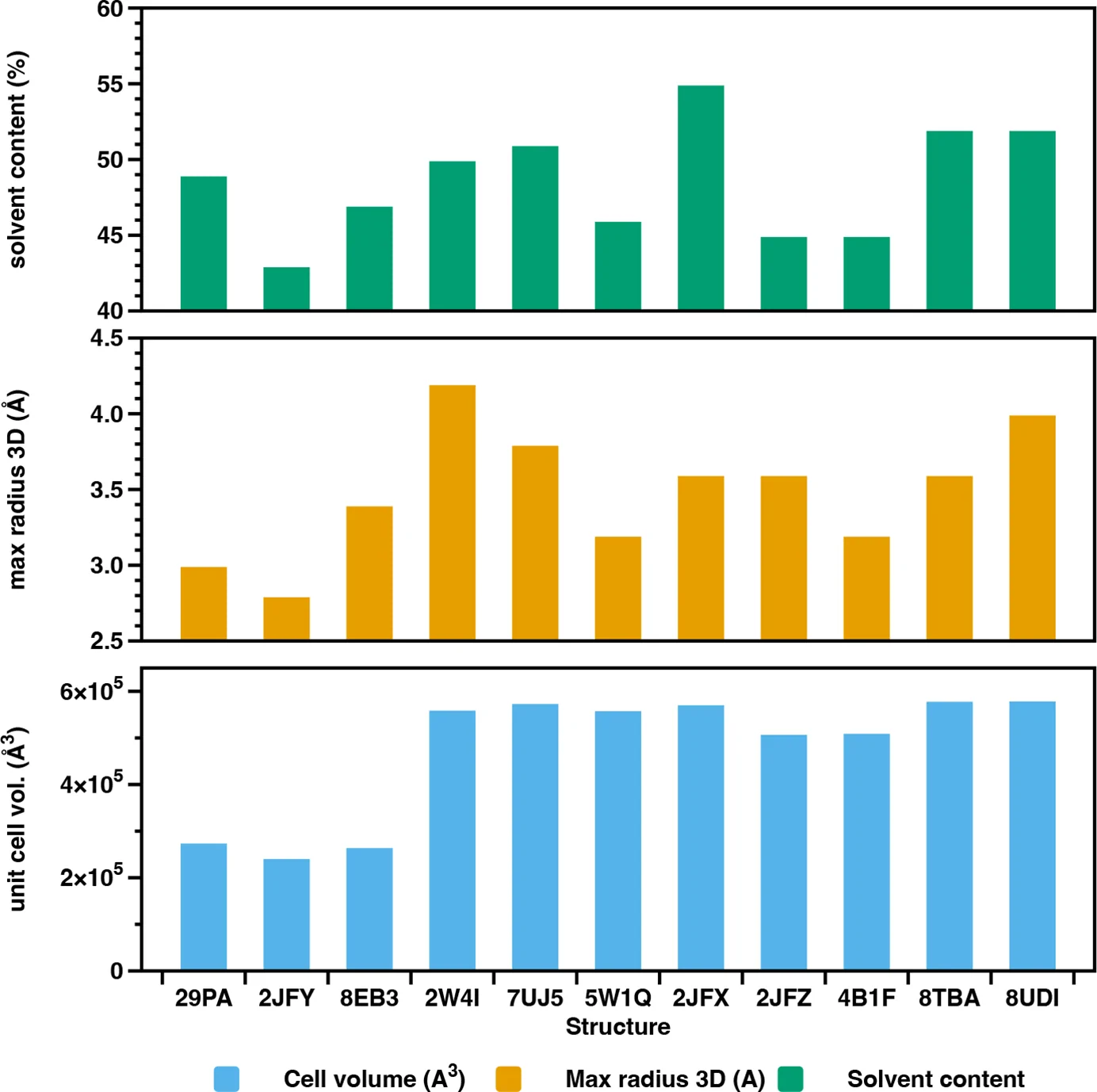
Comparison of the solvent content, maximum channel radius and unit-cell volume across *H. pylori* MurI structures.

Solvent-channel analysis was performed using *MAP_CHANNELS* (Juers & Ruffin, 2014 ▸) to evaluate the connectivity of the solvent network within the crystal lattice. Our structure exhibits a maximum three-dimensional solvent-channel radius of 3.00 Å. Comparison with previously reported *H. pylori* MurI crystal forms (Fig. 5 ▸) shows that the optimized crystallization conditions produced a crystal form with solvent characteristics comparable to those of the previously reported *P*2_1_ structures with one dimer in the asymmetric unit, while yielding improved crystal morphology and higher diffraction resolution. Together with the homogeneous morphology and small size of crystals obtained through optimization of the crystallization conditions, these solvent-channel characteristics make the present crystal form suitable for future mixing-based time-resolved crystallography experiments, where efficient ligand diffusion throughout the crystal lattice is desirable (Schulz *et al.*, 2022 ▸).

**Figure 6:**
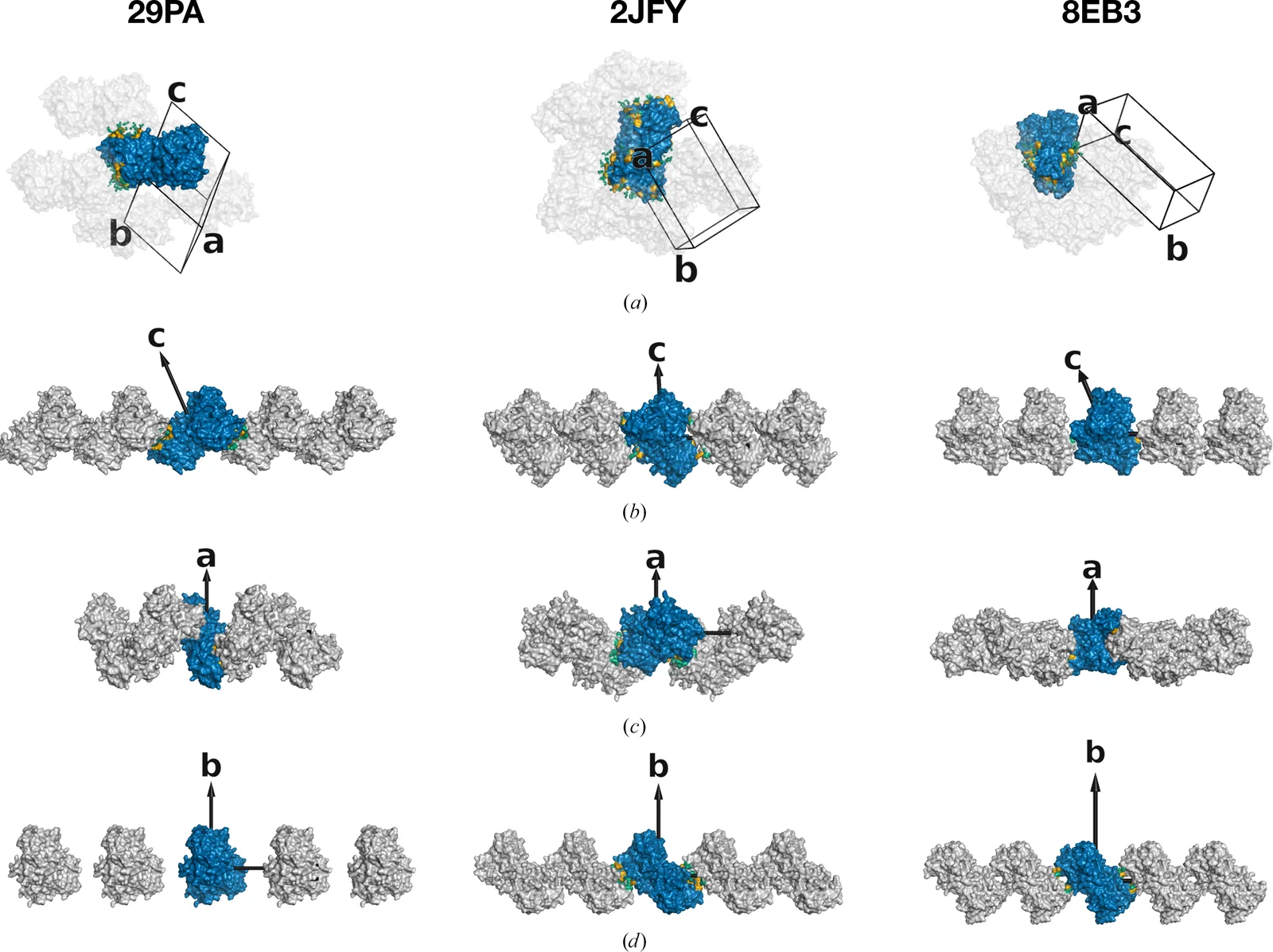
Crystal-packing comparison of PDB entries 29pa (space group *P*2_1_; *a* = 59.1, *b* = 73.0, *c* = 70.2 Å, β = 113.6°), 2jfy (space group *P*2_1_; *a* = 52.3, *b* = 79.0, *c* = 59.1 Å, β = 92.6°) and 8eb3 (space group *P*2_1_; *a* = 53.0, *b* = 96.0, *c* = 57.1 Å, β = 113.0°). For each structure, (*a*) a three-dimensional view of the lattice contacts within the unit-cell and slices of one-dimensional arrays of molecules along the (*b*) *a*, (*c*) *b* and (*d)c* axes are shown. In every panel the reference asymmetric unit is shown in blue and symmetry-related molecules in grey. Interacting residues at crystal contacts are drawn as spheres: orange for the reference molecule and green for the symmetry-related molecule. Crystallographic axes are indicated by black arrows labelled **a**, **b** and **c**.

Crystal-packing arrangements were subsequently compared using symmetry-related intermolecular contacts generated from the deposited crystallographic models (Fig. 6 ▸). For the refined structure with PDB code 29pa, the distribution of symmetry-related contacts and the resulting packing organization across the three crystallographic planes are illustrated in Supplementary Fig. S4. The symmetry-related contacts identified in PDB entry 29pa are summarized in Table 4 ▸, providing a description of the intermolecular interactions contributing to the observed packing arrangement at a residue level.

This comparison revealed that PDB entries 29pa, 2jfy and 8eb3 have similar one-dimensional arrays of MurI dimers. In PDB entries 2jfy and 8eb3 these arrays extend along the crystallographic *c* axis, whereas in PDB entry 29pa they extend along the *a* axis. This apparent difference is a consequence of the unit-cell definition for the monoclinic *P*2_1_ symmetry, with *a* < *c* < *b*, in which alternative choices of the unit-cell axes may describe equivalent packing features.

While the packing interactions within the one-dimensional arrays of dimers could be similar, the relative arrangement of neighbouring arrays differs between the crystal forms. These differences give rise to distinct lattice organizations, unit-cell parameters and crystallographic symmetry descriptions, despite preservation of the MurI dimeric assembly. Extension of the comparison with the remaining deposited *H. pylori* MurI structures (Table 3 ▸; Supplementary Fig. S5) demonstrates that variability among crystal forms primarily reflects alternative organization of MurI dimers within the crystal lattice rather than changes in the protein quaternary structure. These observations indicate that optimization of the crystallization conditions generated a crystal form with improved diffraction properties while preserving the conserved MurI dimeric assembly within a distinct crystal-packing arrangement.

Taken together, the packing analysis indicates that the crystallographic environment can accommodate substantial variability while preserving the structural integrity of the MurI dimer. The occurrence of related dimer-array arrangements in different crystal forms, combined with differences in how these arrays associate with one another, illustrates the plasticity of intermolecular organization within the crystal lattice. This distinction between variability at the crystal-contact level and conservation of the protein structure is further supported by the low C^α^ r.m.s.d. values observed across the available MurI structures, including structures belonging to different space groups. From this perspective, the crystal packing represents an important source of structural variability at the lattice level, while having a comparatively limited influence on the overall MurI architecture. The potential relationship between specific packing contacts and local conformational features, including the cryptic allosteric region described previously (Lundqvist *et al.*, 2007 ▸), remains an interesting question for future comparative structural studies.

## 4. Conclusions

In the present study, the crystal structure of *H. pylori* MurI is presented in monoclinic symmetry, confirming the dimeric assembly in the asymmetric unit and the conserved active-site architecture in comparison with previously reported models for the same species.

Comparative analysis of crystal packing reveals that the conserved dimeric assembly can adopt diverse lattice arrangements, with some crystal forms exhibiting recurring one-dimensional dimer packing features. Variations in the organization of these assemblies contribute to differences in crystal packing and unit-cell parameters.

The crystallization screening yielded crystal forms with differences in solvent content and lattice organization. These observations highlight the influence of crystallization conditions on crystal packing and structural variability, with seeding contributing to improved homogeneity and modulation of crystal morphology while preserving the overall protein architecture. In addition to facilitating structure determination at 1.43 Å resolution, the resulting crystal form provides a promising starting point for future time-resolved crystallo­graphic studies. The combination of homogeneous crystal morphology, small crystal size and high diffraction quality is particularly advantageous for mixing-based experiments, where rapid and reproducible ligand diffusion throughout the crystal population is required. A more comprehensive assessment of the suitability of different crystal forms for time-resolved studies, including the effects of crystal size and lattice organization on diffusion properties, could be part of future studies.

## Supplementary Material

PDB reference: MurI, 29pa

BioRxiv preprint.: https://doi.org/10.64898/2026.04.02.716094

## Figures and Tables

**Table 1:** Data-collection and refinement statistics for MurI Values in parentheses are for the highest resolution shell.

PDB code	29pa
Data collection	
Source	P13, EMBL
Beam size (µm)	70 × 70
Flux (photons s^−1^)	4.97 × 10^11^
Energy (keV)	11.7
Exposure time (ms)	8
No. of images	3600
Oscillation range per image (°)	0.1
Space group	*P*2_1_
*a*, *b*, *c* (Å)	59.09, 72.95, 70.16
α, β, γ (°)	90, 113.59, 90
Resolution range (Å)	64.29–1.43 (1.59–1.43)
Total reflections	460372 (20886)
*R*_meas_	0.119 (1.371)
*R*_p.i.m._	0.044 (0.524)
Unique reflections	64959 (3248)
Mean *I*/σ(*I*)	9.1 (1.4)
Completeness (ellipsoidal) (%)	87.4 (40.9)
Multiplicity	7.1 (6.4)
CC_1/2_	0.998 (0.558)
Refinement	
Resolution range (Å)	48.23–1.43
*STARANISO* ellipsoidal resolution limits	1.42 along 0.83a* − 0.55b*, 1.87 along b*, 1.50 along 0.17a* + 0.99c*
No. of reflections	64951
*R*_work_	0.1543
*R*_free_	0.1958
Wilson *B* factor (Å^2^)	18.30
Mean *B* factor (Å^2^)	
Overall	25.32
Protein	24.60
Water	33.99
Ligands	14.09
Model quality	
Ramachandran plot	
Favoured (%)	98.22
Allowed (%)	1.78
Outliers (%)	0.00
R.m.s.d.s	
Bond lengths (Å)	0.015
Bond angles (°)	2.06

**Table 2:** Summary of crystal morphologies and unit-cell observations for *H. pylori* MurI

pH	PEG [%(*w*/*v*)]	Seeded	Crystal morphology	*a*, *b*, *c* (Å), β (°)	Solvent (%)
7.0–7.5	10–15	No/yes	Long, thick needles	—	—
8.0–8.5	10–15	No/yes	Long, thin needles	59.19, 108.90, 96.23, 100.17	54.1
7.0–7.5	20–25	No/yes	Long, thin needles	—	—
8.0–8.5	20–25	No	Short, thick needles	59.25, 73.80, 68.76, 110.52	50.0
8.0–8.5	20–25	Yes	Small cubes	59.09, 72.95, 70.16, 113.59	49.4

**Table 3:** Crystal and asymmetric unit properties for *H. pylori* MurI structures

PDB code	Space group	Resolution (Å)	*a* (Å)	*b* (Å)	*c* (Å)	α (°)	β (°)	γ (°)	*V* (Å^3^)	*N*_sym_	*n*_chain_	Asymmetric unit volume (Å^3^)	Solvent content	*V*_M_ (Å^3^ Da^−1^)	Maxiumum radius 3D (Å)
29pa	*P*2_1_	1.43	59.1	73.0	70.2	90.0	113.6	90.0	277141	2	2	138571	0.49	2.43	3.0
2jfy	*P*2_1_	1.90	52.3	79.0	59.1	90.0	92.6	90.0	243872	2	2	121936	0.43	2.14	2.8
8eb3	*P*2_1_	2.20	53.0	96.0	57.1	90.0	113.0	90.0	267335	2	2	133668	0.47	2.30	3.4
2w4i	*P*2_1_	1.87	59.1	94.0	101.3	90.0	91.5	90.0	562196	2	4	281098	0.50	2.46	4.2
7uj5	*P*2_1_	1.75	62.0	86.3	107.7	90.0	91.6	90.0	576428	2	4	288214	0.51	2.51	3.8
5w1q	*P*22_1_2_1_	1.95	61.9	85.6	106.0	90.0	90.0	90.0	561103	4	2	140276	0.46	2.29	3.2
2jfx	*P*2_1_2_1_2_1_	2.30	62.2	81.1	113.8	90.0	90.0	90.0	573759	4	2	143440	0.51	2.51	3.6
2jfz	*P*2_1_2_1_2_1_	1.86	61.4	76.3	108.9	90.0	90.0	90.0	510421	4	2	127605	0.45	2.24	3.6
4b1f	*P*2_1_2_1_2_1_	2.05	61.9	76.0	108.9	90.0	90.0	90.0	512572	4	2	128143	0.45	2.25	3.2
8tba	*P*2_1_2_1_2_1_	1.65	63.4	79.9	114.9	90.0	90.0	90.0	581287	4	2	145322	0.52	2.54	3.6
8udi	*P*2_1_2_1_2_1_	2.10	62.3	82.0	113.9	90.0	90.0	90.0	581997	4	2	145499	0.52	2.56	4.0

**Table 4:** Crystal contacts identified using symmetry-related molecules in PDB entry 29pa

Chain 1	Residue/atom 1	Chain 2	Residue/atom 2	Symmetry operation	Cell	Distance (Å)
*B*	Glu62 OE2	*A*	Tyr 217 OH	−*x*, *y* −1/2, −z	212	2.54
*A*	Gln189 NE2	*B*	Asn127 OD1	−*x*, *y* + 1/2, −*z* + 1	223	2.59
*B*	Pro60 O	*A*	Glu94 OE2	−*x*, *y* − 1/2, −*z*	212	2.62
*A*	Thr230 OG1	*B*	Glu246 OE2	*x* − 1, *y*, *z*	122	2.65
*B*	Asn227 O	*A*	Lys250 CE	*x* + 1, *y*, *z*	322	2.65
*A*	Thr203 OG1	*B*	Ser138 OG	−*x*, *y* + 1/2, −*z* + 1	223	2.66
*B*	Lys42 NZ	*A*	Val37 O	*x*, *y*, *z*	222	2.78
*B*	Thr230 OG1	*A*	Phe236 CE1	*x* + 1, *y*, *z*	322	2.80
*B*	Glu235 OE2	*A*	Lys101 NZ	−*x*, *y* − 1/2, −*z*	212	2.82
*A*	Pro202 O	*B*	His139 N	−*x*, *y* + 1/2, −*z* + 1	223	2.83
*A*	Lys42 NZ	*B*	Val37 O	*x*, *y*, *z*	222	2.85
*A*	Glu192 OE1	*B*	Lys130 NZ	−*x*, *y* + 1/2, −*z* + 1	223	2.86
*B*	His61 CE1	*A*	Gln131 O	−*x*, *y* − 1/2, −*z*	212	2.86
*B*	Ser143 OG	*A*	Glu151 OE2	*x*, *y*, *z*	222	2.86
*B*	Ala35 O	*A*	Lys42 NZ	*x*, *y*, *z*	222	2.93
*A*	Ala35 O	*B*	Lys42 NZ	*x*, *y*, *z*	222	2.94
*B*	Glu246 OE2	*A*	Thr230 N	*x* + 1, *y*, *z*	322	2.95
*A*	Pro202 O	*B*	Ser138 CA	−*x*, *y* + 1/2, −*z* + 1	223	3.01
*A*	Pro202 O	*B*	Ser138 CB	−*x*, *y* + 1/2, −*z* + 1	223	3.06
*A*	Phe231 O	*B*	Lys233 NZ	*x* − 1, *y*, *z*	122	3.07
*B*	Lys233 NZ	*A*	Cys229 SG	*x* + 1, *y*, *z*	322	3.11
*A*	Lys117 NZ	*B*	Glu151 OE2	*x*, *y*, *z*	222	3.16
*B*	Asn227 O	*A*	Lys250 NZ	*x* + 1, *y*, *z*	322	3.16
*A*	Glu79 OE2	*B*	Lys59 NZ	−*x*, *y* + 1/2, −*z*	222	3.19
*B*	Glu235 OE1	*A*	Lys101 NZ	−*x*, *y* − 1/2, −*z*	212	3.19
*A*	Asp241 N	*B*	Asp43 OD1	*x*, *y*, *z*	222	3.20
*B*	Lys117 NZ	*A*	Glu151 OE1	*x*, *y*, *z*	222	3.20
